# Tulving’s (1989) Doctrine of Concordance Revisited

**DOI:** 10.5334/joc.447

**Published:** 2025-05-23

**Authors:** Bennett L. Schwartz, Anne M. Cleary

**Affiliations:** 1 Florida International University, US; 2 Colorado State University, US

**Keywords:** confidence, conscious experience, subjective experience, consciousness, déjà vu experience, dual process theories of recognition, Doctrine of Concordance, experience, metacognition, remember/know judgments, retrospective confidence

## Abstract

The Doctrine of Concordance is the implicit assumption that cognitive processes, behavior, and phenomenological experience are highly correlated (Tulving, 1989). Tulving challenged this assumption, pointing to domains in which conscious experience did not accompany a particular measured cognitive process and to situations in which consciousness did not correlate with the observable behavior. Schwartz (1999) extended this view, asserting that the underlying cognitive processes that produce conscious experience may differ from those that produce observable behavior. Though research on conscious experience blossomed during the last quarter century and progress has been made in moving past the Doctrine of Concordance, we argue that some subdomains within memory research remain hampered by an implicit endorsement of it. We outline two areas of memory research in which current research and interpretations appear to fall prey to the Doctrine today: research on the dual- vs. single-process theory in recognition memory, including work on remember/know judgments, and research on retrospective memory confidence. We then describe four areas of research that show progress in understanding conscious experience by rejecting the Doctrine of Concordance: These are 1) metacognitive disconnects in the science of learning, 2) recognition illusions, 3) déjà vu experiences, and 4) aha experiences. We claim that there is often a dissociation between the mechanisms that create conscious experience and the underlying cognitive processes that contribute to behaviors, which may seem causally correlated with conscious experience. Disentangling the relations between process, behavior, and conscious experience in the human mind’s operation are important to understanding it.

## Background and Introduction

Tulving (1989) argued that consciousness in memory research was an understudied area, and that this understudy needed to be addressed by researchers. In Tulving’s view, memory research was still stunted by the lingering influence of behaviorism and the verbal learning era. This influence led to cognitive scientists’ timidness in challenging the assumption that cognitive processes, conscious experience, and behavior are highly correlated and largely mappable to one another, a view he called the Doctrine of Concordance. Tulving thought that this state of affairs discouraged research on conscious experience itself. Thus, Tulving’s 1989 paper was a call to emphasize the importance of conscious experience in memory.

Today, there is a thriving body of research on issues of consciousness and conscious experience, including on the neural mechanisms that correlate with consciousness and the potential functions of evolved consciousness (Dehaene & Naccache, 2001; Lau & Rosenthal, 2011; Mudros et al., 2025; Persuh, 2018; Walter, 2021). In the domain of human memory, the focus of this paper, this includes an extensive literature on subjective judgments of memory experience, such as remember-know judgments (Geraci et al., 2009; Umanath et al., 2023; Yonelinas, 2002) and on the notion of mental time travel (Greenspan & Loftus, 2024; Schacter et al., 2017). The research concerning memory and consciousness also includes an extensive literature on metamemory, that is, our awareness of our own mnemonic processes (Dunlosky & Metcalfe, 2009; Efklides, 2011; Koriat, 2024; Umanath et al., 2025; Undorf et al., 2018). These literatures expand every day at a rate faster than any individual person can keep up with, and we applaud this work. This focus and attention on memory and consciousness is, in our view, a strong positive for the field of cognitive psychology, and it represents an advance from the state of the field when Tulving wrote his paper on the Doctrine of Concordance (Tulving, 1989).

In this review paper, we revisit Tulving’s Doctrine of Concordance to examine progress made since the time of Tulving’s (1989) article as well as to assess in what ways present day research is still shadowed by the assumptions of the Doctrine of Concordance. Our view is that, despite the new world in which there are journals, conferences, and societies devoted to the study of consciousness and metamemory, the field still has not released itself fully from the cage that the Doctrine created, and this has hindered research progress across areas that address conscious experience, especially in the domains of memory and metamemory. In many areas of study of the study of memory, despite the explicit attention to conscious experience, the assumptions of the research still reflect the Doctrine Concordance. Despite the progress that has been made in the study of conscious experience since Tulving’s challenge to the Doctrine Concordance, his points continue to have a meaningful message to today’s researchers.

We also will describe Schwartz’s (1999) extension of the Doctrine of Concordance, which was made with Tulving’s full assent (also see Neisser et al., 2023). Although Tulving limited himself to observing that consciousness was not always correlated with cognitive processes and behavior, Schwartz argued that consciousness and behavior may arise from separate cognitive processes. We describe areas of research in which the Doctrine of Concordance is challenged either explicitly or implicitly and how this leads to a better understanding of the role of phenomenology in human memory. We also will describe domains of research that, despite the content of their area being about conscious experience, the research focus and assumptions still conform to the Doctrine of Concordance. The areas we criticize for their adherence to the Doctrine are dual-process theory within the study of recognition memory, including remember/know judgments, and retrospective confidence judgments. The specific areas that we praise for challenging the Doctrine include research metacognitive disconnects in learning science, illusions of recognition, the déjà vu experience, and the aha experience in insight problem-solving. We argue that conscious experience is important to study on its own for the sake of understanding human consciousness, and that we are more quickly moving toward a better understanding of conscious experience in those areas in which the Doctrine of Concordance has been challenged than in areas in which it has not been challenged. We further make a plea that more theorists and researchers today explicitly acknowledge Tulving’s challenge to the Doctrine of Concordance in other areas in which the Doctrine has not been challenged as much, and we suggest that to better understand the basis of conscious experience itself, researchers should aim to delineate the interrelations between basic cognitive processes, overt behaviors, and conscious experience.

## The Doctrine of Concordance

In 1989, Endel Tulving wrote the first paper for the first issue of the first volume of the *European Journal of Cognitive Psychology* (now the *Journal of Cognitive Psychology*). In this paper, Tulving criticized a concept he labeled the Doctrine of Concordance. In Tulving’s view, the Doctrine of Concordance is the implicit assumption that cognitive processes, behavior, and conscious experience are highly correlated. That is, if we examine the relation between the cognitive process and the behavioral consequences, then we can also predict the conscious experience. In Tulving’s words, the Doctrine means that there is a “general, if not perfect, agreement between what people know, how they behave, and what they experience” (Tulving, 1989, p. 8). Under the doctrine, cognitive processes mean the mental computations, behavior are the observable actions, and conscious experiences are the qualia that accompany cognition. Thus, for example, if we understand how prior experience affects recognition judgments, we can make inferences about familiarity processes and performance, but we can also talk about familiarity experiences and performance. Tulving claimed that it is the implicit Doctrine of Concordance that led cognitive psychologists to pay insufficient attention to the conscious experiences^1^ that accompany memory. Thus, he described the Doctrine of Concordance in order to criticize it and replace it with an approach to memory that embraced the importance of studying conscious experience itself.

Tulving thought the human mind worked differently than the traditional view reflected in Concordance, and that conscious experience mattered (Tulving, 1989). It was his experience as a scientist that conscious experience does not always correlate with the cognitive processes of memory, and that both may not be always correlated with behavior. As such, he challenged this generally-held view of a tight correlation (or concordance) between the three entities. To be clear, Tulving claimed that the Doctrine of Concordance was a long-held implicit assumption in psychological science, but he thought it was an assumption that needed to be questioned and overturned. Thus, he advocated against this Doctrine not for it. This needs to be clear as the term itself, the Doctrine of Concordance, was a term he coined. Thus, his initial goal in his 1989 paper was to demonstrate domains in which the conscious experience did not correlate with objective behavior or with the inferred underlying cognitive processes.

Tulving (1989) described a number of areas – from implicit memory to source amnesia—in which the correlation between cognitive processes and conscious experiences was weak. Although this may seem obvious now, Tulving argued that mental computations (that is, cognitive processes) are not identical to conscious experience. For example, in terms of memory research, the cognitive process of retrieval (or ecphory in Tulving’s jargon) is not equivalent to the conscious experiences of recollection. Retrieval can happen with or without conscious experiences (McDermott & Roediger, 1993; Roediger & McDermott, 1993), and in some cases, conscious experience of memory can occur without retrieval (e.g., Cleary et al., 2012). Tulving (1983) argued that recall is based on a retrieval process which combines external cues with pre-existing memory representations. This yields a particular response but may or may not yield a conscious experience. There are instances in which objective recollection appears to have occurred, but the conscious experience may be lacking, as in the case of a person who, after brain injury, could recollect experiences without feeling a sense of personal ownership of them (Klein & Nichols, 2012). Another example occurs with amnesic patients, who show evidence of implicit memory but may lack the conscious experience of recollection. In this case, a dissociation between the cognitive process of retrieval, present in implicit memory, and the lack of a conscious experience of recollection contradicts the Doctrine of Concordance. For another example, Tulving pointed to remember/know judgments in which recognition performance may be strong regardless of whether the accompanying conscious judgment is ‘remember’ or ‘know.’ That is, the behavior – correctly choosing the old item is present, regardless of whether the accompanying conscious experience is a ‘remember’ feeling or a ’know’ feeling. Conversely conscious experiences of memory may also occur in the absence of retrieval. For example, Huebert et al. (2022) explore situations in which there is a feeling of recollection when objective recollection has not occurred. To summarize, Tulving’s challenge to the Doctrine of Concordance is that there are situations in which behavior, cognitive processes, and conscious experience are uncorrelated.

After private discussions with (and with written endorsement from) Endel Tulving, Schwartz (1999) expanded on Tulving’s challenge to the Doctrine of Concordance (Tulving, 1998, personal communication to BLS). In Tulving’s original paper, Tulving did not specify why cognitive processes and conscious experience are not always correlated. That is, he does not propose a psychological or neurological explanation by which behavior, cognitive processing, and conscious experience are related in some cases, and in some cases uncorrelated.

**Figure 1:**
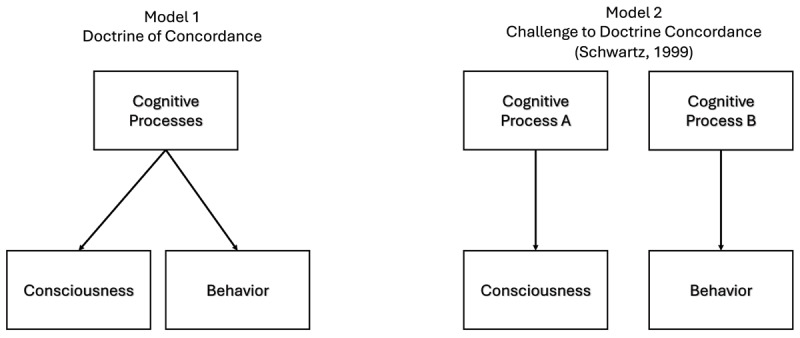
Model 1 shows a conceptualization of the Doctrine of Concordance. The same cognitive process is responsible for both consciousness and behavior, leading to a strong correlation between cognitive processes, consciousness, and behavior. Model 2 shows the challenge to the Doctrine of Concordance elaborated by Schwartz (1999). In it one cognitive process leads to conscious experience, whereas a second cognitive process leads to observable behavior. This general model can account for why we observe dissociations between cognitive processes, consciousness, and behavior.

While reviewing the existing literature on the tip-of-the-tongue phenomenon (henceforth, TOT), Schwartz (1999) claimed that a potential reason for the breakdowns in concordance is that one set of cognitive processes may underlie retrieval, and a second, potentially different set of cognitive processes may underlie the conscious experiences, such as TOTs, associated with retrieval (Schwartz, 1999; see also Cleary et al., 2025) (see Figure 1). Already by the late 1990’s, our research was showing dissociations between recall and TOT states (e.g., Metcalfe et al., 1993; Schwartz & Smith, 1997). The failure of the retrieval process may lead someone to be unable to recall a certain word, but a different cognitive process leads to the accompanying TOT (Schwartz, 1999; Umanath et al., 2025). To be more specific, retrieval failure may be caused by the lack of access to the lexeme (Gollan & Brown, 2006), but the experience of the TOT can be caused by factors such as the familiarity of the cue and the presence of retrieved related information (Cleary, 2019; Ryals et al., 2021; Schwartz & Smith, 1997; Schwartz & Metcalfe, 2011). Thus, rather than arguing there is a lack of correlation between cognitive processes and conscious experience, we expanded the criticism of the Doctrine of Concordance to claim that, in some cases, the processes underlying object-level performance (e.g., recall) and the processes underlying meta-level monitoring (TOTs) were different and dissociable (see Koriat, 1993; Metcalfe, 1993). In the metacognition literature (e.g., Nelson & Narens, 1990), object-level refers to the cognitive processes taking place “under the hood” to keep us going, such as encoding, retrieving, or attending to particular things in the environment. In contrast, meta-level refers to the processes that allow us to introspect on or reflect on aspects of those object-level cognitive processes that are available to conscious introspection. Some of these meta-level processes produce conscious experience. In this paper, although we acknowledge the seminal importance of Tulving’s conception of the challenge to the Doctrine of Concordance, we expand it to include instances in which the cognitive processes that produce object-level processes and behavior are different from the cognitive processes that produce conscious experience.

## Why Tulving Invoked the Doctrine of Concordance

Tulving (1989) invokes the “standard fable” (p.5) of a time when behaviorists were dominant. In the fable of the behaviorist view, only observable behavior matters and to even speak of issues of either cognitive process or conscious experience is to deviate from good science. As cognitive psychology emerged from behaviorism, it became acceptable to discuss issues of cognitive processes and issues of representation. With the beginning of cognitive psychology, behavior serves the function of revealing that which is hidden, namely the underlying largely non-conscious sets of process that control our behavior. Thus, even when conscious experience is involved, such as it is in visual imagery, the research infers a cognitive process, such as how we match geometric figures from reaction time behavior (e.g., Shepard & Metzler, 1971). Though the data here match conscious experience (that is, it feels like we are doing mental rotation), it is the behavioral data that drove conclusions, not conscious experience. This was a big step forward, but in Tulving’s view, still absent from this view is the acceptance that conscious experience matters and that to be complete psychological scientists, we must understand it as well. He described progress in understanding the nature of underlying cognitive processes and proposed that we also start attending to issues of consciousness in memory. To summarize Tulving’s view, he wrote “…I see little explicit concern with conscious awareness in remembering (p.7)”. This was both a concern to him and a challenge to make things different. The Doctrine of Concordance was Tulving’s explanation for why there was such little research on conscious experience – it was not necessary because it merely reflected that which was easier to measure in the first place, the encoding and retrieval behavior of people.

**Figure 2:**
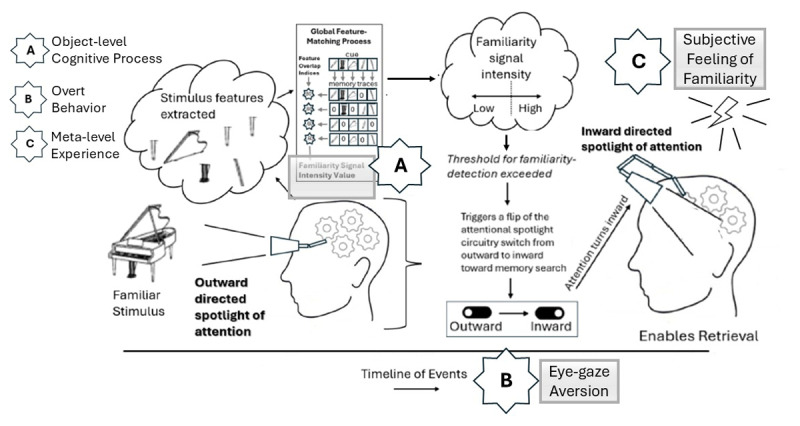
Illustration of a hypothetical situation in which an object-level basic cognitive process, an overt behavior, and a meta-level conscious subjective experience are interrelated, yet each based on a different underlying mechanism. Here, the hypothesized meta-level conscious experience of a sensation of familiarity (C) arises from a different mechanism than the object-level process of familiarity signal computation (A) and there is not direct conscious access to the output from (A). Figure adapted from Figure 2 of Cleary et al. (2025), an illustration of the familiarity-flip-of-attention theory. A) depicts the object-level familiarity signal intensity value output from the global-feature-matching-based familiarity signal computation specified in the MINERVA 2 model (Hintzman, 1988). B) depicts an overt behavior: Eye-gaze aversion—an indicator of shutting out visual inputs to focus attention inward toward memory (Servais et al., 2023). C) Depicts a meta-level subjective conscious experience: The feeling of familiarity; in this case, the feeling of familiarity results not from direct access to the familiarity signal intensity value output depicted in A), but rather from conscious detection of the fact that one’s attention is inward-focused on searching memory for something that has yet to come to mind.

Note, of course, that Tulving’s (1989) view does not preclude the possibility that often there may be concordance between process, consciousness, and behavior. Indeed, it is easy to generate many situations in which they do, as in the above example about mental rotation (Shepard & Metzler, 1971). Our primary thesis here is that fully understanding the human mind will require determining in which situations object-level and meta-level operations differ from one another and in what ways they differ. In some instances, they may have different bases (see Figure 1). In other instances, object-level and meta-level operations may be interrelated in complex ways that do not entail a one-to-one mapping (see Figure 2). As a case in point, Kirwan et al. (2009) demonstrated that neural responses exhibited patterns consistent with memory for recently studied information even when overt behavioral responses did not, suggesting that the neural mechanisms of memory differentiation differed from overt behavioral responses. As another case in point, Cleary et al. (2025) proposed a complex cascade of events for specifying how fast-acting object-level mechanisms of familiarity signal computation might ultimately relate to the conscious experience of familiarity (see Figure 2). Fully understanding these complex interrelations—including in which situations object-level and meta-level mechanisms overlap and in which situations they diverge—will be critical to unraveling the puzzle of how human conscious experiences arises from basic mechanistic processes.

Cleary et al.’s (2025) proposal for how an object-level memory mechanism (familiarity signal computation) might contribute to a conscious experience of memory (a subjective feeling of familiarity) serves as an example of the type of theoretical research that we argue needs to be done to better and more often to understand the nature of conscious experience. As depicted in Figure 2, Cleary et al. proposed that the global-feature-matching mechanism purported to underlie familiarity signal computation in global matching models of recognition memory might serve as an attentional modulator within the larger cognitive system. Specifically, Cleary et al. posited that when the feature-matching based familiarity signal exceeds an established internal criterion, it serves as a gating mechanism that flips attention from outward-directed toward stimuli in the environment to inward-directed toward a search of memory representations. That is the object-level mechanism that leads to a whole suite of behaviors. For example, one potential consequence of attention flipping from outward-directed to inward-directed is that a person’s behavior may then change in measurable ways; for instance, some researchers have noted that when the mind turns inward, people shut out external visual inputs, which is detectable through measuring eye gaze behavior (Salvi et al., 2015; Servais et al., 2023). Note here that the object-level cognitive mechanism (global-feature-matching-based familiarity signal computation; e.g., Hintzman, 1988) is distinguishable from the observable behavior (eye gaze shifting). In addition, the meta-level conscious experience of familiarity is distinguishable from both the observable behavior and the cognitive mechanism underlying that behavior. Specifically, Cleary et al. suggest that the conscious experience of familiarity arises when the experiencer reflects that their attention is inward-directed in memory search mode and failing to retrieve relevant information. In this way, the conscious experience of familiarity is based on a different specific mechanism (i.e., noticing that one is stuck in memory search mode without a retrieved product from memory) than the mechanism that drove the familiarity signal computation within the cognitive system (i.e., global-feature-matching-based familiarity signal computation). This is an example of a theoretical approach that does not assume the Doctrine of Concordance. In contrast, an approach that implicitly accepts the Doctrine of Concordance is one in which the conscious experience of familiarity is itself the output of the global-feature-matching-based familiarity signal computation; that is, the familiarity signal intensity that is the output of this computational mechanism is itself accessible to consciousness to produce the feeling and its intensity. As we describe below, many uses of formal memory models involve this implicit assumption.

Moving forward from 1989 to 2025, we see a massive change in the attention to consciousness, conscious experience, and phenomenological experience on the part of cognitive psychology. For example, there are multiple journals in existence whose primary purpose is to support and disseminate new scientific data on consciousness. As one example, there is the journal “Consciousness and Cognition,” which started publishing papers in 1992 shortly after Tulving’s 1989 paper. There is also the APA journal, “Psychology of Consciousness: Theory, Research, and Practice,” which has been published since 2013. In 2015, the journal “Neuroscience of Consciousness” sprung into existence. The open-access Frontiers journals have a ‘section’ entitled “Consciousness Research.” Thus, the world has changed with respect to the study of consciousness since Tulving wrote his Doctrine of Concordance paper back in 1989. Why, then, the need to revisit the Doctrine?

First, although Tulving’s research has changed the field of memory in many ways including the introduction of episodic memory and the ideas of accessibility and availability (e.g., Tulving, 1983), for the most part the Doctrine of Concordance has not received as much attention as other Tulving papers have, although we believe it should. According to Google Scholar on December 27, 2024, Tulving’s 1989 paper has only been referenced 364 times. To some this may seem like a lot, but some of Tulving’s papers have tens of thousands of references. Second, the idea of challenging the Doctrine of Concordance has guided much of the first author’s research (Schwartz, 1999; 2002; Schwartz & Metcalfe, 2011). Third, and most importantly, despite the much greater attention to issues of consciousness and phenomenological experience in contemporary Cognitive Psychology, we argue here that even when researchers are explicitly interested in what conscious experience is, their approach and interpretations of their data are often still influenced by the Doctrine of Concordance. That is, even work directly developed to tell us something about conscious experience may, as we will see, be used as evidence for the way in which a particular object-level cognitive process works rather than understanding the conscious experience itself as separable from object-level cognitive processes. As such, in the next 6 sections, we will explore examples of well-studied conscious experience and assert that some of the research is still based on assumptions that the processes that underlie cognition are the same as those that underlie conscious experience.

We start by considering two examples in which research on meta-level conscious experience has been conflated with understanding the object-level cognitive processes of memory. These are the dual- vs. single-process debate in recognition memory, including remember/know judgments, and retrospective memory confidence judgments. In these cases, some research has either implicitly inferred conscious experience from measured object-level cognitive processes or has employed meta-level judgments of conscious experience as a way of examining underlying object-level memory processes without considering that the object-level processes that produce overt behavioral responses on a cognitive task may not map directly onto a meta-level conscious experience and vice versa (e.g., Cleary et al., 2025; Kirwan et al., 2009), thus illuminating the continued influence of the Doctrine of Concordance.

We then consider four domains in which the research has sought to examine the mechanisms underlying the meta-level conscious experience itself without assuming equivalence with object-level processes: 1) research examining a metacognitive disconnect between impressions of learning and learning outcome, 2) memory illusions, 3) the déjà vu experience, and 4) the aha experience. These four research domains have involved a focus on conscious experience itself as potentially different from underlying object-level cognitive processes or overt behaviors, thus representing the kind of progress that we believe Tulving (1989) was calling for when raising the issue of the Doctrine of Concordance.

## Areas Still in Need of Progress Separating Conscious Experience from Cognitive Processes

### The Dual-Process Debate in Recognition Memory and Remember-know Judgments

One area still in need of major progress in moving away from the Doctrine of Concordance is the research that concerns the dual-process debate in the study of recognition memory. The dual-process debate revolves around whether old-new discrimination in standard recognition memory paradigms is the result of a single process (such as that which is described by signal detection theory and models that are derived from it) or by two different processes (familiarity, which is the ability to sense that something is familiar in the absence of an ability to recollect specifics, and recollection, which is the ability to call to mind specific details about a prior experience). Unfortunately, in much of the literature concerning the dual-process debate, object-level memory processes and meta-level conscious experiences of memory are conflated. Moreover, researchers who study this issue tend to see conscious experience as a direct correlate of the underlying cognitive process.

In day-to-day life, people clearly have subjectively different experiences from feeling as if something seems familiar without being able to pinpoint why to calling to mind the specific details of a prior experience. Starting with Tulving (1985), we have a paradigm for addressing these different conscious experiences, namely the remember/know procedure. Indeed, some of the debate in the study of dual process vs. single process theory in recognition memory revolves around how to interpret remember/know judgments. It is our view that this debate is based on an implicit endorsement of the Doctrine of Concordance. Our assertion is that research on remember/know judgments is better considered from the perspective of how conscious experiences arise from underlying process that differ from the processes that enact recognition rather than using remember/know judgments to determine if recognition involves one process or two. This view stands in contrast to most work on remember/know judgments in the last few decades.

Remember/know judgments have their origins in Tulving’s initial empirical work (Tulving, 1985), and he explicitly addressed them in the 1989 Doctrine of Concordance paper (Tulving, 1989). In his view, the then nascent work on remember/know judgments concerned the study of the phenomenological experience of recognizing information that was stored in memory (Gardiner, 1988; Gardiner & Java, 1990; Rajaram, 1993; Tulving, 1985). Indeed, according to Tulving, ‘remember’ judgments reflect autonoetic consciousness (the sense of self moving through past, present, and future), whereas ‘know’ judgments reflect noetic consciousness (the sense of just knowing something, like a fact). Recently, De Brigard (2024) argues that evidence does not strongly support a distinction between noetic consciousness and autonoetic consciousness, but Tulving’s approach to remember/know judgments is not wedded to one particular view of conscious experience. Rather, these judgments were intended, by Tulving, to represent the reporting of a person’s conscious experience, regardless of what the underlying causes were. Understanding what led to ‘remember’ and ‘know’ judgments, then is about exploring the nature and causes of differences in conscious experience. For Tulving, the finding of a dissociation between remember and know judgments and variables such as level of processing show that we can subjectively distinguish unique states of conscious experience and that these experiences do not have a one-to-one relation to memory processes.

However, despite the initial interest in conscious experience, the Doctrine of Concordance is strong in us memory researchers.^2^ It did not take long for the focus on conscious experience to be distracted by the use of remember/know judgments to explore underlying mechanisms of object-level memory processes. Research in remember/know judgments quickly morphed from being about the nature of meta-level conscious experience during memory retrieval to delineating different processes responsible for that retrieval (e.g., Starns & Ratcliffe, 2008; Yonelinas, 2002). Wixted and Mickes (2010) wrote, for example, that “the use of the remember/know procedure evolved and became more commonly used in relation to the dual-process theory of recognition memory.” (p. 1027). That is, counter to the original intention of Tulving, Wixted and Mickes report that almost all research on remember/know judgments is not to investigate the nature of conscious experience, but to examine dual-process models of recognition, in which remember judgments are thought to be a function of the object-level process of recollection, and know judgments arise from the object-level process of familiarity. Wixted and Mickes note that frequently in the literature remember/know judgments are also used to reveal strong versus weak memories that derive from a single process, their preferred view. Thus, despite Tulving’s optimism, the work on remember/know judgments has been re-absorbed into a tradition that assumes that conscious experience arises from the same processes as memory retrieval. However, we stress the fact that people have subjectively distinguishable metacognitive experiences that they can associate with these terms is interesting in its own right to study, and it does not necessarily map onto underlying mechanisms of familiarity signal computation versus successful retrieval of content. Thus, our claim here is the Doctrine of Concordance has influenced much of the recent work on remember/know judgments. Rather than approach the topic of remember/know judgments as a topic of interest because conscious experience is of interest, it has been re-cast to explore underlying processes of memory performance.

Umanath and Coane (2020), like Wixted and Mickes (2010), showed that most research on the remember/know judgments comes from the perspective of differentiating dual processes of recollection and familiarity. In fact, Umanath and Coane (2020) report that 95% of the 899 studies they entered into their review were designed to test various aspects of the dual process theory of recognition. The remainder used remember/know judgments to differentiate between episodic or semantic memory or to differentiate the Wixted and Mickes perspective that remember/know judgments reflect differences in memory strength along a single continuum. First, note that they collected 899 studies that used remember/know judgments. Thus, these were a large number of researchers attracted to looking at conscious experience, but they did so with the intent of studying cognitive processes, conflating memory processes and conscious experience. Thus, consistent with the arguments made here that the Doctrine of Concordance is still implicitly endorsed, what Umanath and Coane (2020) show is that nearly 100% of the papers done on remember/know judgments use the judgments to explore underlying cognitive processes of memory rather than trying to determine what cognitive processes underlie the conscious experience that remember/know judgments embody. Thus, even today when considering explicit measures of conscious experience, research tacitly assumes the Doctrine of Concordance—that subjective metacognitive experiences of memory reflect their underlying cognitive processes.

In their second study, Umanath and Coane (2020) examined how both experts and non-experts define the experience of remembering and the experience of knowing. In contrast to experts, most non-experts produce definitions that accord with Tulving’s (1985) original conception of the difference between remember and know judgments, namely that the feeling of remembering mirrors what happens when we retrieve information from episodic memory but that the feeling of knowing mirrors what happens when we retrieve information from semantic memory. This stands in contrast to the typical use of remember/know judgments to differentiate recollection and familiarity processes. Umanath and Coane (2020) represents the rare study in which the focus is not on what remember/know judgments tell us about underlying processes of memory, but what is the nature of the phenomenological experience (also see the work of Gardiner, 1988; Gardiner & Java, 1990; 1993). We think that the study of remember/know judgments would benefit from an explicit challenge to the Doctrine of Concordance. Variables should be investigated that influence the balance of remember/know judgments that do not affect recognition performance, such that we can understand the processes that contribute to remember/know judgments that do not contribute to recognition performance. In metacognition work, this dissociation technique has been successful at teasing apart the processes that produce metacognition. For example, in feeling-of-knowing judgments, variables have been tested that affect recall without affecting the feeling-of-knowing judgments, and variables have been tested that affect feeling-of-knowing judgments without affecting recall (Metcalfe et al., 1993). This logic should again be applied to remember/know judgments to determine what processes produce the judgments themselves, similar to the early work on the topic by Gardiner and by Rajaram (1993).

### Retrospective Confidence

Retrospective confidence, also known as post-answer confidence, refers to the conscious experience concerning the level of certainty that retrieved information is accurate. Thus, if someone asks for the name of the capital city of Jamaica, and you retrieve “Kingston,” your retrospective confidence judgment indicates how certain are you that you are correct. Thus, a person may respond “Kingston” and be correct, but show low confidence, whereas another person may respond “Montego Bay” and be incorrect, but show high confidence. Often the goal of research on retrospective confidence judgments is to demonstrate if it is accurate at predicting performance. In memory research, retrospective confidence judgments are directed at retrieval from of various forms of memory, including episodic memory, eyewitness memory, and working memory (e.g., Conte et al., 2023; Delay & Wixted, 2021; Koriat, 2024; Pournaghdali et al., 2025; Wixted et al., 2015; Wixted & Wells, 2017). In a typical experiment on retrospective confidence, participants are asked to retrieve information, either information learned in the experiment or general knowledge. Once that information is retrieved, participants are asked to judge the likelihood that the retrieved information is correct. These retrospective confidence judgments can be measured for accuracy against the actual correct answer. In most cases, retrospective confidence judgments accurately reflect the likelihood that the retrieved information was correct (Tekin & Roediger, 2017).

In recent work, we argued that most explanations of retrospective confidence focus on direct-access theories (Schwartz, 2024). Direct-access theory, in metacognition, means that the same processes that produce object-level performance, such as retrieval, also drive meta-level performance, such as retrospective confidence. That is, retrospective confidence judgments are made based on the strength of retrieved information (see Bjork & Bjork, 1992 for different approaches to memory strength). Though it need not be, we suspect that many direct-access approaches to metacognitive judgments come from implicit endorsements of the Doctrine of Concordance. That is, there is a tendency to believe that the same processes that produce the object-level memory also produces the accompanying conscious experience associated with the metacognitive judgment. Models such as those developed by Fleming (2024) and Delay and Wixted (2021) use retrieval strength as the main basis of retrospective confidence. Indeed, in both the Fleming (2024) and Delay and Wixted (2021) other potential causes of retrospective confidence are dismissed as ‘bias’ rather than as a real cause of retrospective confidence. Both models are quite detailed and informed by much data in the field. However, we use them as examples of models that implicitly endorse the doctrine of concordance and as a consequence disregard data that the conscious experience of retrospective confidence does not always directly result from the retrieval process.

Delay and Wixted (2021) presented two models to account for the relation between retrospective confidence and memory accuracy. The first model is based on signal detection, and the second model is a threshold model. Both models begin with the assumption that retrospective confidence judgments are based on the strength of the memory (an assumption that dates back to early models of memory, including global matching models of recognition memory; Clark & Gronlund, 1996). Although there is much research to support this assumption, there is also research that points to other factors that are equally important in driving retrospective confidence (e.g., Koriat, 2024). In Delay and Wixted, in contrast, factors not directly tied to the strength of the retrieved memory, such as retrieval fluency, are considered factors that influence the criterion at which people set different confidence levels, rather than directly influencing the experience of retrospective confidence. Thus, the main factor in retrospective confidence is retrieval strength. Similarly, Fleming’s (2024) model dismisses factors other than memory strength as noise and then uses memory strength to predict retrospective confidence. Our point here is not that these models are not useful in understanding and predicting retrospective confidence, but rather at their heart, they implicitly endorse the Doctrine of Concordance, in that they largely assert that conscious experience (retrospective confidence) follows directly from object-level cognitive processes (retrieval).

In fact, research demonstrates that a number of factors influence retrospective confidence judgments in addition to memory strength (see Schwartz, 2024, for a review). This research shows that there is a causal relation between several non-retrieval factors and retrospective confidence judgments. These causal factors include the fluency of the cue or question pointing to the retrieved answer, the fluency by which the answer is retrieved, domain familiarity (how knowledgeable the person is about the topic), beliefs about the retrieval process or the specific answer, and the consensuality or belief that others would give the same answer (Alter & Oppenheimer, 2009; Brewer & Sampaio, 2006; Koriat, 2012; 2024; Shaw, 1996). For example, Shaw (1996) showed that the post-event questioning resulted in higher confidence for final retrospective confidence judgments, despite the fact that the questioning did not change final performance on a recognition test. In another study, Fiechter and Kornell (2021) found that repeating the question increased the retrospective confidence judgments, without affecting recall of those answers on the final test. In both cases (and many more) researchers have shown that retrospective confidence judgments are influenced by a host of processes other than the strength of the retrieval (Schwartz, 2024). Thus, there is ample evidence to suggest that some of the mechanisms that produce retrospective confidence judgments are different from the mechanisms that produce retrieval. This leads us to a challenge to the Doctrine of Concordance. The processes that underlie retrieval are not identical to the processes that underlie retrospective confidence judgments, consistent with Tulving’s challenge (1989).

To summarize, the disconnect between the existing models of retrospective confidence judgments and the data are illuminated by the Doctrine of Concordance. Implicitly, there is an assumption that the meta-level process that produces metacognition (or conscious experience) is the same as the process that directs the object-level task performance, namely retrieval. The Doctrine of Concordance results in the thinking that there is a direct relation between retrospective confidence judgments and the strength of the retrieval. Reflecting the Doctrine, most current models consider the main influence of confidence to be strength and other factors to be noise (cf., Koriat, 2024; Schwartz, 2024). However, when we challenge the Doctrine, we see that one set of object-level cognitive processes is responsible for retrieval, but a partially non-overlapping set of processes is responsible for the conscious experience, in this case, the retrospective confidence judgments. Thus, further advances in the area of retrospective confidence would be improved by directly considering the implicit endorsement of the Doctrine of Concordance and how explanations would improve if we rejected the Doctrine.

## Areas Showing Progress in Separating Conscious Experience from Cognitive Processes

Not all domains of cognitive psychology continue to implicitly endorse the Doctrine of Concordance. In fact, many domains have made progress in understanding conscious subjective experience by rejecting the Doctrine of Concordance. Below, we describe four areas of research in which progress has been made by moving away the Doctrine.

### Metacognitive Disconnects Shown in the Science of Learning

One area that shows great progress in moving away from the idea that the processes behind conscious experiences map directly onto measured memory processes can be found in the science-of-learning literature. Over approximately the past 15 to 20 years, researchers examining strategies for enhancing learning in educational settings have uncovered many instances in which people’s conscious experience of their learning is dissociable with their actual learning outcomes. In fact, it is now well-established that people’s conscious experience of their learning processes does not map onto their actual learning processes (e.g., Deslauriers et al., 2019; Kirk-Johnson et al., 2019; Rhodes et al., 2020).

The metacognitive disconnect between conscious experiences of learning and learning itself has been shown in several ways. One such disconnect concerns the spacing effect. The spacing effect, which is the finding that distribution of learning episodes across time leads to better learning than massing learning episodes together, has been established since the time of Ebbinghaus (Ebbinghaus, 1885; 1964) and widely replicated across time (Greene, 2008; Hintzman, 1974) as well as shown to be a highly generalizable principle of learning (e.g., Baddeley & Longman, 1978; Hartwig et al., 2022; Kang, 2017; Kapler et al., 2015; Rohrer et al., 2015; Seabrook et al., 2005). However, research over the past 15 years or so has demonstrated a pervasive disconnect between people’s conscious experience of what spacing is doing for their learning and their actual learning. For example, Kornell and Bjork (2008) demonstrated that although interleaving of specific to-be-remembered examples of paintings by a particular artist among other artists’ paintings led to better inductive learning of an artist’s genre of painting, participants’ judgments of their learning were the opposite: People consistently judged that they were learning better with the massed format than with the spaced format. As another example, Kornell (2009) found that although spaced repetition of flashcards led to better learning, participants consistently had the conscious experience during learning that massing was better for their learning. This metacognitive disconnect regarding spacing and interleaving harkens back to an early hint of such a disconnect first shown by Baddeley and Longman (1978), who found that training postal workers in the skill of typing using different training schedules led to a similar metacognitive disconnect, whereby the most distributed spacing schedule resulted in the best skill learning yet the conscious experience of learning was the opposite: People felt most satisfied with the most massed schedule and felt the least satisfied about the most spaced schedule. This literature demonstrates a clear example of a situation in which conscious experience does not map onto the underlying cognitive processes that underlie actual learning. As Cleary and Rhodes (2023) and Rhodes et al. (2020) note, there is a disconnect between people’s subjective impressions of their learning and what is actually going on “under the hood”, so to speak. The underlying learning mechanisms benefit from spacing, whereas the conscious subjective metacognitive experience of the learning process emerges from something else (not the actual learning process). That is, one process is creating the conscious experience, whereas another is leading to the memory behaviors.

As to what that “something else” might be that is responsible for the conscious experience of learning, some clues suggest that fluency of processing might be a culprit. In another study to demonstrate a disconnect between conscious impressions of learning and actual learning outcomes, Benjamin et al. (1998) asked participants to answer general knowledge questions as quickly as they could, pushing a button as soon as they had the answer. Upon producing the answer, the participant had to judge the likelihood that they would be able to recall that answer later on when given a free recall test. The faster they could answer the initial question, the more likely participants thought they would recall it later on the free recall test. However, the opposite was true: The longer it took to answer the question, the more likely it was that the answer would be recalled on the free recall test. In this case, participants’ conscious experience of memorability seemed to be based on the ease with which a piece of information could be accessed from memory in that moment, or its momentary fluency/accessibility, whereas the actual memory retention mechanism itself had a different basis, namely the distinctiveness of the target answer. Massing may similarly result in high momentary feelings of fluency/accessibility compared to spacing, resulting in a similar disconnect.

The testing effect is another example of a case in which a metacognitive disconnect between conscious experiences of learning and the mechanisms that underlie learning itself has been shown. The testing effect is the highly replicable and pervasive finding that retrieving information from memory leads to better memorability of that information than restudying it (Rowland, 2014). That is, spending time retrieving the information from memory facilitates later memorability compared to merely being re-presented with the information (Abbott, 1909) and thus testing is a useful strategy for enhancing learning outcomes. However, as with the spacing effect described above, people’s conscious experiences of learning are at odds with this reality about the actual learning process. For example, in their study examining vocabulary learning comparing testing vs. restudying, Kornell and Son (2009) found the usual testing effect (testing led to better later retention than restudying) but found that learners’ judgments of their own learning were higher during restudying than during testing. Others have similarly shown that people are generally consciously unaware of the benefits of testing to their learning while the testing is taking place (e.g., Roediger & Karpicke, 2018). As with spacing, this disconnect might be because of the conscious experience of in-the-moment fluency or accessibility or the lack thereof, as testing can create an in-the-moment feeling of *lack of* accessibility whereas restudying can potentially afford a high sense of fluency and accessibility. This is another clear example of a case in which conscious experience does not map onto cognitive processes taking place “under the hood” so to speak, illustrating the importance of Tulving’s points about the Doctrine of Concordance.

One final example in the realm of the science of learning concerns learning styles. Learning Style is the idea that everyone has a particular “style” of learning (e.g., Cleary & Robinson, 2025; Dekker & Kim, 2022; Robinson et al., 2022), such as being primarily a visual, auditory or kinesthetic learner, and that learning will be best when the teaching method matches the style of the learner (e.g., Rhodes et al., 2020). A common example is that visual learners learn best when material is presented visually, auditory learners learn best when materials are presented verbally or auditorily, and kinesthetic learners learn best when performing actions on materials (e.g., Rhodes et al., 2020). The scientific consensus is that this concept of Learning Styles has no merit (Cleary & Rhodes, 2023; Cleary & Robinson, 2025; Dekker & Kim, 2022; Knoll et al., 2017; Pashler et al., 2008; Robinson et al., 2022; Willingham et al., 2015). Still, people’s conscious experiences of their own learning during the learning process are disconnected from that reality. Knoll et al. (2017) found that people believed themselves to be learning better when information was presented in their preferred mode of receiving information (visual or verbal) even though the mode of receiving information did not actually matter to the retention of the information. Knoll et al. argued that these beliefs led to higher judgments of learning, which led people to believe they were more likely to remember information in a consistent learning style. Moreover, learning in a preferred style may result in more fluent processing, leading to higher expectations of learning, without any actual memory advantages. That is, from other situations we know that fluent processing may create the illusion of better learning even in situations in which it actually results in weaker memory performance (Benjamin et al., 1998). This suggests yet another example of conscious experience not mapping onto the cognitive processes taking place “under the hood.” However, researchers keep returning to this issue for another look because of the pervasive belief that conscious experiences of learning should correlate with learning (Knoll et al., 2017).

### Recognition Memory Illusions

Somewhat similar to the disconnects shown in the science of learning literature are disconnects shown in the recognition memory literature in the form of illusions (or biases) using recognition memory paradigms. In a typical recognition memory paradigm, participants first study a list (or lists) of items and are then given a recognition test containing a mixture of old (studied) and new (unstudied) items, and the task is to discriminate old from new items. Many mathematical models of recognition memory exist, and most are variants of signal detection theory (e.g., Clark & Gronlund, 1996; Hintzman, 1988; Wixted, 2007; Wixted & Mickes, 2010), which separates old-new discriminability from criterion placement. As described above in the section in which continued progress on advancing from the Doctrine of Concordance is needed, some illusory forms of recognition memory are commonly dismissed in memory research as bias (or criterion placement) when the focus is on real memory discrimination and are, as a consequence, often not the primary focus of study when the interest is in the memory mechanisms. However, we argue that many of the biases commonly found in recognition memory research are phenomena that are indicative of conscious experiences of memory that are therefore worthy of study on their own and as separable from the cognitive processes that underlie memory retention itself.

One such illusion of memory is the recognition memory bias discovered by Jacoby and Whitehouse (1989), which provided an initial clue that the processes that give rise to conscious experiences of memory and the processes that give rise to memory retention itself are not the same. Jacoby and Whitehouse presented participants with a study list of words followed by a recognition test containing a mixture of old (studied) and new (unstudied) words. On the test, the test words were preceded by a rapidly-flashed masked prime word. Some of the time, the rapidly-flashed masked prime word was identical to the soon-to-follow recognition test word; some of the time, it was a different word. In addition, some of the time the rapidly-flashed masked prime word was presented too quickly for conscious identification of the word and some of the time, it was presented for a long enough duration to be consciously perceived. Participants exhibited an increased tendency to call the full-view target test word old if it was preceded by a rapidly-flashed masked copy of itself than if it was preceded by a rapidly-flashed masked presentation of a different word. This pattern only occurred at the more rapid, less perceptible prime durations and not at the slower more perceptible durations of prime words. The pattern suggests that the increased perceptual fluency brought on by the matching, rapidly-masked prime words felt like oldness (or like the *conscious experience* of recognition memory) to the participants. In this way, this particular illusion of memory is indicative of a fundamental difference between the cognitive processes that produce actual memory performance (i.e., behavior) and those that produce the conscious experience of memory. The non-mappability of the processes that drive actual memory and the processes that drive the conscious experience of memory help to drive home Tulving’s (1989) points about the Doctrine of Concordance.

Another recognition bias that disconnects memory processes from subjective memory experience is the revelation effect (Peynircioglu & Tekcan, 1993; Watkins & Peynircioglu, 1990; see Aßfalg et al., 2017, for a review). The revelation effect is the finding that, when recognition test items need to be revealed as opposed to just being presented, such as when solving the test word from an anagram, the revealed items are more likely to be called “old” than items presented intact. This bias occurs even when a counterfeit study list is used in which participants are told that they are receiving a difficult-to-see study list when in actuality, no study items were presented in the study phase (Frigo et al., 1999), powerfully demonstrating the disconnect of conscious experience of recognition from the cognitive processes that underlie memory for study list items.

Yet another recognition bias that disconnects actual memory processes from conscious memory experience is the pseudoword effect (Greene, 2004). The pseudoword effect is the finding that pseudowords (pronounceable nonwords) consistently receive more “old” judgments on recognition memory tests than words. Greene (2004) argued that this bias is because the pseudowords are more familiar in recognition memory paradigm contexts than actual words. This idea might at first seem to be at odds with another recognition bias—the word frequency bias (Miller, 2010)—but might actually be compatible with it. Miller (2010) found that when a counterfeit study list was used (in which participants were told that they were receiving a difficult-to-see study list when in actuality, no test items were presented during the study phase), participants exhibited a bias toward calling high frequency recognition test words old relative to low frequency words. This contrasts with the usual word frequency mirror effect that is found when an actual study list is used, and it suggests that when no actual memory for study list items is possible and participants are left with only the possibility of biases, high word frequency is subjectively experienced as memory for study list items. This fits with Greene’s (2004) explanation for the pseudoword effect insofar as certain stimulus characteristics can *feel like* the kind of familiarity that emerges from test items familiarized from an earlier study list. In turn, this reveals something about the mechanisms of conscious experience that is separable from memory mechanisms themselves.

Similar to the aforementioned recognition test biases is the buzzing chair bias discovered by Goldinger and Hansen (2005). In a unique analog to what Jacoby and Whitehouse (1989) did, Goldinger and Hansen had participants study a list of words, then during the recognition test, had the chair on which the participant was sitting buzz a barely perceptible buzz underneath the person. Participants exhibited a bias analogous to the Jacoby-Whitehouse effect in which recognition test items accompanied by a barely perceptible sensation of a buzz from underneath the chair were more often judged to be “old” than items unaccompanied by the buzz. Like the Jacoby-Whitehouse effect, when the buzz was more obvious and clearly perceptible, the bias went away. Thus, like the Jacoby-Whitehouse effect, the pseudoword effect, and the word frequency bias, the buzzing chair bias reveals something about the conscious experience of memory as separable from the mechanisms responsible for actual memory. What all of these aforementioned biases seem to have in common is that they result from what feels like a subjective sensation of familiarity—a sense of fluency that is not easily attributable to a source other than the study list can *feel like* familiarity from recent list exposure. Likewise, a barely perceptible buzzing can *feel like* familiarity from a recent list exposure.

An interesting case of a conscious experience that may *feel like* familiarity from a recent list exposure without being due to that memory representation is the tip-of-the-tongue state (TOT), which is the feeling of being on the verge of accessing a word. Cleary (2006) found that being in a TOT state was associated with stronger feelings of oldness on a recognition test. Participants studied a list of words that were each an answer to a general knowledge question that would appear later on at test. At test, participants attempted to answer each question, indicated if they were feeling a TOT state for the word, and rated the likelihood that the word had appeared on the immediately preceding study list. When participants were in a TOT state for an unretrieved target word, they gave significantly higher ratings of the likelihood that the unretrieved word was studied than when they were not in a TOT state, and this pattern occurred even for unstudied words. Thus, participants exhibited a TOT-state bias whereby TOT states subjectively felt like the unretrieved word was presented recently on a study list. This represents another case in which the conscious experience of memory does not map onto the processes that underlie actual memory retention or discriminability. This particular bias may suggest something about the phenomenology of both the sense of oldness in an old-new recognition memory paradigm and the sense that a word is on the tip of the tongue.

Not every type of recognition memory bias is in the form of a mistaken feeling of list-based familiarity, however. One puzzling bias that may reveal something important about the conscious experience of memory is the materials-based bias effect discovered by Lindsay and colleagues (Fallow & Lindsay, 2022; Lindsay & Kantner, 2011; Lindsay et al., 2015). They found that on recognition tests, participants are consistently more conservative in their recognition responses when the materials are images of paintings than when the materials are words. When lists are blocked (i.e., all of a kind), participants are neutral in their response bias to words but conservative in their response bias to images of paintings (responding “old” less often). When lists are mixed (i.e., a mixture of words and images of paintings), participants are more liberal with word stimuli, responding “old” more often to words. The precise reason for the materials-based bias is unclear but represents yet another case in which the experience of memory does not map onto the processes responsible for memory retention or discriminability.

Another memory bias that is not in the form of a mistaken feeling of list-based familiarity is familiarity-driven recollective confabulation. The concept of familiarity-driven recollective confabulation was first suggested by Moulin (2018) when considering why some clinical patients who experience frequent seizure-related déjà vu also confabulate reasons for their déjà vu experience. Moulin suggested that the strong sensation of familiarity might drive a need for explaining the feeling away, such as by conjuring potential reasons through illusory recollective experience. He further conjectured that familiarity-driven recollective confabulation might be a part of the normal operation of everyday memory and suggested that future research investigate that possibility.

To test this idea, Huebert et al. (2022) carried out a study to investigate this possibility in a list-learning recognition memory paradigm. After studying a list of words, participants were presented with a list of nonword test cues, each of which potentially resembled a segment of the study list to a varying degree. Based on prior theory-driven research on the recognition without cued recall phenomenon (e.g., Ryals & Cleary, 2012), a cue that resembles four different unrecalled studied items will feel more familiar than will a cue that resembles only one studied item that fails to be recalled; in turn, a cue that resembles only one studied word that fails to be recalled will feel more familiar than a cue that resembles no studied items. This pattern fits global matching models of recognition memory in which having a higher degree of global feature-match to studied items generates a stronger familiarity signal. Huebert et al. used this cue familiarization approach to show that as the experimentally-induced feelings of familiarity with the cue were systematically increased (through the global feature overlap manipulation during instances of cued recall failure), there was a corresponding increase in false recollective experience for an experimental detail that was actually not objectively recollected. That is, the more familiar-seeming the cue, the more participants erroneously believed that they could produce an episodic detail that had accompanied the cue’s unrecalled target word. This familiarity-driven false recollective experience points toward another way in which conscious memory experience does not map onto actual memory retention or discriminability mechanisms. Like the other recognition memory biases discussed here, familiarity-driven recollective confabulation is another metacognitive disconnect from underlying memory mechanisms that is worthy of study in its own right for what it might reveal about conscious experience. Finally, familiarity-driven recollective confabulation also presents another reason why Tulving’s (1989) points about the Doctrine of Concordance are so important today and why cases in which researchers continue to make implicit assumptions suggesting adherence to a Doctrine of Concordance continue to need to be challenged; that is, there needs to be an ongoing separation of memory mechanisms that produce memory retention and discriminability and mechanisms that produce the conscious metacognitive experience of memory.

### The Déjà vu Experience

Another example of a research domain in which significant progress has been made on escaping the Doctrine of Concordance is the study of the déjà vu. The déjà vu experience is defined as the strong feeling that something we are experiencing now has been experienced before, despite the knowledge that is not the case (Cleary & Brown, 2021). Déjà vu experiences are rare, but most people have experienced them, and they may be more common in people who suffer from certain forms of epilepsy (Cleary & Brown, 2021). We discuss the déjà vu experience here because it is a strong conscious experience of remembering, but one that is not always tied to a specific object-level piece of information or to specific memory process. That is, déjà vu experiences are linked to memory, but the specific nature of déjà vu experiences is that they cannot always be tied to a specific memory in the way that TOTs are specific to a particular word (Schwartz & Cleary, 2016). Thus, in many ways, they represent a phenomenon in which conscious experience can be studied because we want to understand the nature of the conscious experience and the processes that underlie that experience rather than what conscious experience tells about behavioral outcomes such as recall or recognition and the processes that underlie retrieval (e.g., McNeely-White & Cleary, 2019). As such, research on the déjà vu experience focuses on what causes the *experience* rather than what it tells us about halted or misguided retrieval.

Cleary et al. (2012) hypothesized that the déjà vu experience arose from an underlying process of familiarity that ultimately leads to a conscious feeling of familiarity. The familiarity process prompts people to feel like the experience they are having now is something they have experienced before, regardless of the actual source of the higher level of familiarity (e.g., see Figure 2). In keeping with the current concerns, Cleary et al. proposed a mechanism for why we have déjà vu experiences rather than the processes that lead us to resolve high-familiarity, low-recollection memory situations. Note that in this view, whether the feeling of familiarity is justified by an actual memory experience or if a retrieval process ultimately produces the missing context is irrelevant. What is important is that an underlying cognitive process produces a conscious experience, which may or may not be correlated or in common with the processes that produce an actual memory experience. Thus, Cleary et al. (2012) passes the bar of challenging with the Doctrine of Concordance.

In their empirical work, participants first visited a series of virtual-reality scenes. For example, the participants might visit a bowling alley or a main street in a small town. In Cleary et al. (2012) participants passively viewed the scenes through the VR headsets, though in later work, they actively moved through them (e.g., Cleary et al., 2021a; Okada et al., 2024). After completing a set of visits to virtual-reality scenes, the participants later returned to the virtual-reality world and viewed new scenes. Some of these scenes had an identical geometrical layout to a scene seen earlier. That is, in the second trip through the virtual-reality world, a participant might visit a church that has the same geometry as the bowling alley or a city scape that has the same geometry as the main street in the small town. The similar geometry was designed to allow for an increase in familiarity for those scenes that had a spatial layout match in the earlier phase. The question that Cleary et al. (2012) asked was if this increased experimental familiarity would boost the probability of reported déjà vu experiences. Indeed, it did. People reliably reported more déjà vu experiences for scenes about which they had earlier seen a scene with similar geometry but that failed to be recalled. Thus, Cleary et al. (2012) showed that déjà vu experiences are at least partially based on a cognitive process of familiarity, or that the process of detecting familiarity can contribute to a déjà vu experience. Much work since has replicated this basic finding (see Cleary et al., 2021a; Cleary & Brown, 2021; Okada et al., 2024).

Of particular interest to our discussion of the Doctrine of Concordance, Cleary and Claxton (2018) carried out a study in which they examined a possible mechanism for the association between déjà vu and the sense of knowing what is going to happen next. They had participants view a series of virtual tours from the first-person perspective, then had them engage in a test in which they were taken on a new series of virtual tours, also from the first-person perspective. Half of the tours were of novel scenes that mapped onto study phase scenes in spatial layout and movement trajectory; half were of novel scenes that did not map to studied scenes. Cleary and Claxton set out to examine the hypothesis that déjà vu’s association with feelings of prediction might be grounded in the same unrecalled memory that contributes to boosting the probability of experiencing déjà vu in the first place. They specifically examined whether, upon stopping short of a turn taken in a similarly configured scene toured earlier, people would exhibit an increased ability to predict the direction of the next turn in the test scene and if so, if this would be associated with feelings of déjà vu. However, they found no such association. During instances of recall failure and reports of déjà vu, people were at chance in their ability to predict the direction of the next turn. However, in follow-up experiments, Cleary and Claxton (2018) and others (Cleary et al., 2021a) demonstrated that despite actual memory-based predictive ability being at chance during recall failure, déjà vu was associated with strong illusory *feelings* of prediction. This illusory sense of prediction that accompanies the déjà vu experience represents another case in which the processes that cause conscious experience are separable from the cognitive mechanisms producing actual memory retention ability or performance.

Moreover, feelings of déjà vu themselves also occur for idiosyncratic reasons that are not due to the experimental familiarization manipulation of scene spatial layout. Cleary et al. (2019) followed-up on the consistent pattern that although the probability of reporting déjà vu is significantly boosted by experimental spatial layout familiarization, the probability is still higher than zero among scenes in the unfamiliarized condition of these studies, suggesting that although the probability of déjà vu can be boosted through experimental scene familiarization, the feeling of déjà vu is also driven by other idiosyncratic factors not related to the experimental manipulation. Cleary et al. (2019) capitalized on this to examine déjà vu experiences in cases in which the déjà vu could not be tied to an experimental episode because none of the test scenes actually mapped onto a previously toured scene. They found the usual association between déjà vu reports and feelings of prediction regarding the direction of the next turn. However, even more interestingly, when they then allowed the tour to continue into a turn following the pause and the prompting for the indication of any feeling of prediction, Cleary et al. (2019) showed a postdictive bias whereby during déjà vu, people exhibited a bias toward thinking that the turn had gone exactly the way that they felt it was going to go. That is, despite lacking an ability to actually predict the direction of the next turn, during déjà vu people not only felt as if they could predict the direction of the next turn before going into it but also felt after going into it that they knew all along how it was going to go. Like the feeling of prediction bias that often accompanies déjà vu, this feeling of post-diction bias is also untethered to experimentally induced memory mechanisms, further driving home Tulving’s (1989) points about the Doctrine of Concordance.

In the context of the goals of this paper, the déjà vu experience research is relevant for two reasons. First, it serves as a model for how research on conscious experience should be conducted. The methods are straightforward and involve careful experimental control, important when the measure in question is a completely subjective phenomenon. In this work, we see that underlying familiarity processes are contributing causes of déjà vu experiences and that feelings of familiarity correlate with déjà vu experiences, which in turn is related to but separate from a drive to pursue a recollective process which may eventually resolve the déjà vu experience (e.g., McNeely-White & Cleary, 2023). Second, because the goal of Cleary’s research is to understand a purely phenomenological experience, the approach challenges the Doctrine of Concordance. The déjà vu experience is not simply a correlate of some memory process—it is produced by its own unique cognitive underpinnings that are worth trying to understand.

### Insight and the Aha Experience

Consider being given a problem to solve, such as “A landscape gardener is given instructions to plant 4 special trees so that each one is exactly the same distance from each of the others. How is the gardener able to do it? (Experiments 1 and 2, from Metcalfe & Wiebe, 1987).” This problem, and those like it, are considered to be insight problems, as they require a perceptual reorganization to be able to solve. Indeed, as Metcalfe and Wiebe showed, a linear progression leads to failure in insight problem-solving. A sudden perceptual reorganization reveals the answer and, usually, the sudden feeling of insight. For another one, consider “Describe how to put 27 animals in 4 pens in such a way that there is an odd number of animals in each pen.” The cognitive process by which we solve such problems, such as the gardener problem and the animal pen problem, has been well-studied (Grimmer et al., 2023; Wiley & Danek, 2024). Most of us first think of the gardener problem in two-dimensional space, in which there is no solution. As such, our attempt to solve it ends in failure. However, when we reframe the mental image into three dimensions, then we may see the fourth tree atop a small hill (or in a bit of a ditch). Similarly, trying to divide the animals into four separate pens will always end in error. The solution involves encompassing one or more pens into a larger one. As in other domains of cognition, we can consider both how we solve insight problems such as these, and what the conscious experience about those problems is like. Not surprisingly, given the ideas we are developing, you can guess that in some situations, past researchers have assumed that if you understand the process of solving the problem, the feelings of intuitions come directly from that (Weisberg, 2013). That is, our conscious experience of how we solve the problems correlates with the processes by which we solve the problem. Other research has focused on where those feelings of intuition come from and if they are dissociable from the finding of the solution (Collier & Beeman, 2012; Laukkonen et al., 2023; Wiley & Danek, 2024). In the case of insight problem-solving, such as the gardener problem and the animal-pen problem, Metcalfe and Wiebe showed that the confidence in the solution appears in an all-at-once “eureka” experience when the problem is reframed in 3 dimensions. This sudden awareness that one knows how to solve the problem is commonly called the aha experience. Most recently, the AI program Deepseek claims to have aha experiences, indicative of its ability to solve insight problems (Deepseek-AI, 2025).

Wiley and Danek (2024) contrast a traditional view of the aha experience, in which it is something that results directly from sudden cognitive restructuring with an approach in which the aha experience is based on factors other than or in addition to cognitive restructuring. It is this second approach that fits the challenge to the Doctrine of Concordance. That is, aha experiences may arise from different processes from those involved in the cognitive or perceptual restructuring, which allow us to solve the problem. Wiley and Danek point to research that suggests that the increase in fluency that occurs with cognitive restructuring may serve as the basis for the aha experience, similar to the effects of fluency seen in other domains. Changes in emotional state (affect) may do so as well. They also point out that aha experiences may not always correlate with accuracy on the problem-solving task. All of these are reasons to suspect that the processes of restructuring and the aha experience are different, that is, that the processes that produce aha experiences dissociate from problem-solving processes. This dissociation strongly supporting a challenge to the Doctrine of Concordance. Moreover, Wiley and Danek discuss that aha experiences have positive outcomes, such as encouraging curiosity, and in this way map onto other metacognitive phenomena (Cleary, 2019; Metcalfe et al., 2020). As such, the mapping of Wiley and Danek’s view onto the Doctrine of Concordance framework is straightforward. The view that the aha experience reflects cognitive restructuring in insight problem-solving maps onto the traditional view of the relation of process to conscious experience. The second view, and the one preferred by Wiley and Danek, is that the aha experience has different origins than problem-solving processes, and this one maps onto Tulving’s challenge to the Doctrine (Tulving, 1989).

Laukkonen et al. (2023) proposed the existence of an “eureka heuristic.” The eureka heuristic is the combination of the aha experience and its functional utility. That is, the aha experience is part of the heuristic, followed by a metacognitive-control decision as to what needs to follow from the aha experience in order to solve a problem. The aha experience alerts the problem-solver that a solution may exist and the metacognitive-control decision is necessary to follow through with redirecting attention from previous thoughts toward new thoughts that may be more productive in solving the problem. In this way, the aha experience is considered a metacognitive experience and one that might change behavior. The aha-as-heuristic guides behavior that controls problem-solving, rather than the processes that accomplish the problem-solving. Thus, Laukkonen et al. (2023) postulate a separate process for the conscious experience of the aha experience and the object-level problem solving, in keeping with the challenge to the Doctrine. To summarize the logic here, in the insight problem-solving literature, as in many domains of cognitive science, there is interest in the level of the cognitive processes that actually do the problem solving and the feeling of awareness or the “aha” or “eureka” experience that accompanies it (Laukkonen et al., 2023; Metcalfe & Wiebe, 1987; Yaniv & Meyer, 1987). As in many domains in which the Doctrine of Concordance is implicitly endorsed, there continues to be research in which the explanations for the aha experience is considered to be the same as those that drive the problem-solving. For example, Topolinski and Strack (2009) argued that when an insight problem is solved, the solution feels very fluent, leading to an aha experience. In this case, the process that causes the solution to be found is more directly related to the process that drives the phenomenological experience. In contrast, newer views endorse and find evidence to support that different mechanisms underlie the problem-solving and people’s subjective experiences of problem-solving, as in the aha” or “eureka” experience (Laukkonen et al., 2023; Wiley and Danek, 2024).

Interestingly, Laukkonen et al. (2023) describe how, until recently, an ongoing debate in the problem-solving literature was whether insight problem solving occurred via different processes than step-by-step problem solving (see Fleck & Weisberg, 2013).^3^ Early attempts to distinguish them via conscious experience (e.g., Metcalfe & Wiebe, 1987) were dismissed (Weisberg, 2013). It was not until insight and step-by-step problem solving were distinguished by other means, such as their neural signature (Kounios & Beeman, 2014) that the consensus was that they were the result of different processes. Here we see the hidden hand of the Doctrine of Concordance. Conscious experience was assumed to be an epiphenomenon, with no real reality. Only when brain imagery showed different patterns for insight and step-by-step problem solving were they considered the result of separate processes – phenomenological differences are insufficient alone to distinguish them. We are not arguing here that differences in conscious experience always point to differences in underlying cognitive mechanisms, but we do think that the assumed irrelevance of conscious experience leads to its easy dismissal and that needs to be changed.

Laukkonen et al. (2023) show that the processes that lead to the ‘aha’ experience are caused by processes that are separate from and meta- to the processes that do the problem-solving. This separate process has adaptive value – it monitors the ongoing problem solving and provides the person with feedback as to its success, as in their description of the eureka heuristic (also see Valueva et al., 2016; Wiley and Danek, 2024). In this way, the eureka heuristic is similar in its hypothetical adaptivity to the inferential processes that lead to strong confidence after memory retrieval (Schwartz, 2024). Indeed, Laukkonen et al. write “This view of insight as a higher-order representation of coherence or fit also tracks well with other meta-cognitive models….” (page 2).

More support for this approach comes from research on the Presque vu phenomenon, which refers to the feeling of being on the verge of an insight or epiphany. Presque vu means that the conscious experience in question comes before the person solves the problem rather than after the problem is solved. Kostic et al. (2015) found that priming the solutions to analogy problems with their analogy answers in an earlier study list did not boost the probability of solving the problem at test nor did it boost the probability of reporting Presque vu for an unsolved problem at test. However, in a pattern similar to the way that TOT states are associated with subsequent resolution, Kostic et al. found that reported feelings of Presque vu at test were associated with a soon thereafter ability to solve the problem upon being given a second chance. Thus, feelings of Presque vu occurred for idiosyncratic reasons that were not boosted by their list-learning experimental manipulation, but that *were* predictive of arriving at the solution to a problem.

It is as yet unclear if this predictive sense uncovered by Kostic et al. (2015) is related to the aha experience. It is possible that it relates to an understudied phase of insightful problem-solving that Wallas (1926) described as intimation Sadler-Smith (2015). Wallas is known widely for having devised four phases of insightful problem solving: 1) preparation, 2) incubation, 3) illumination and 4) verification. Preparation is initial work on trying to solve the problem. Incubation occurs when one sets the problem aside for a period of time to do something else. Illumination is the “aha!” experience, or the moment of insight. Verification is then the process by which the person goes about determining that the insight experienced in the aha! moment was indeed correct. Sadler-Smith (2015) argue that the creative problem-solving literature has been missing an important component of Wallas’s initial theorizing about insightful problem-solving—a phase that Wallas argued took place between incubation and illumination: intimation. Intimation is the feeling of being close to arriving at a problem’s solution, but that occurs before a solution is reached. We suspect that one reason why the creative problem-solving literature has been avoiding inclusion of Wallas’s stage of intimation is that intimation involves a metacognitive experience that may or may not be directly tied to experimental manipulations or even to eventual arrival at the correct answer.

Dougal and Schooler (2007) found that when participants solved anagrams, they were more likely to attribute the solution of the anagram to an earlier list of words that they had been asked to encode (anagrams are letter strings using the same letters as a target word, such as the word “paroled” is an anagram for “leopard”). This was particularly true for those solutions that were accompanied by an aha experience. In this case the aha experience leads to an inference, in some cases false, that a word had been seen earlier. This finding is possible if we think of the process that produces the aha experience as being an independent feeling that then gets misattributed to the earlier memory list. As we discussed earlier, Cleary and colleagues (e.g., Cleary, 2019; Cleary et al., 2021b) make a similar argument for why TOTs influence judgments about earlier experiences. Thus, to summarize, the research on aha experiences and insight problem-solving has made great strides by working with the idea that the processes that underlie aha experiences are different from those that drive object-level insight problem-solving. We hold up the work on aha experiences as a role model for understanding differences between cognition, including memory, and phenomenology.

## Summary and Conclusions

We have reviewed six areas of conscious experience, remember/know judgments and dual-process theory, retrospective confidence judgments, the metacognitive disconnect in the science of learning, memory illusions, déjà vu experiences, and the aha experience,. In each case, there is a compelling area of study, namely how and why we have these conscious experiences, what mechanisms underlie them, and how they correlate with observable behavior. In each case, we argue that the success of the research program has involved going after what causes the conscious experience and then looking at how the conscious experience predicts performance in cognitive tasks. When the question is about what conscious experience tells us about the object-level behavior, then the results are unclear and less interpretable. We have focused on these examples of conscious experience, but there are others that also fit into this approach. In other work, our colleagues and ourselves have applied the logic of the challenge to the Doctrine of Concordance to the work on TOTs, in which there has long been parallels traditions of research focused on what TOTs tell us about retrieval and what TOTs tell us about conscious experience (see Cleary, 2019; Cleary et al., 2020; Cleary & Schwartz, 2025; Huebert et al., 2023; Schwartz, 1999; Schwartz & Cleary, 2016; Schwartz & Metcalfe, 2011; Schwartz & Pournaghdali, 2021). Other work has looked at the blank-in-the-mind state (Moraitou & Efklides, 2009), and aesthetic chills (Schoeller, et al., 2024), to mention a few conscious experiences that would or do benefit from the challenge approach.

We also claim that the underlying cognitive processes that produce conscious experience may differ from the processes taking place “under the hood” or that cause the memory behavior. We argue that conscious experience is often caused by separate cognitive processes that correlate themselves with the cognitive process that produces the behavior. This is the core extension of Tulving’s (1989) challenge to the Doctrine of Concordance (Schwartz, 1999). Consistent with heuristic views of metacognition, this view is one in which conscious experience evolves/develops separately from underlying cognition, but with the intent to monitor it or reflect on it (Koriat, 1993; Metcalfe, 1993). In our labs, we have found this an important tool in looking at TOTs (e.g., Cleary, 2019; Schwartz & Pournaghdali, 2021).

We think it is important to focus on the conscious experience and how the processes that produce conscious experience work and how they may affect behavior in indirect ways. In particular, Cleary et al. (2023; 2025) focused on the role of conscious experience or metacognitive states in in redirecting or refocusing our attention. For example, they demonstrated that conscious experiences associated with metacognition, although not mappable to underlying processes/mechanisms of memory, might have a broader purpose of helping us to orient our attention to something different than we were, and in ways that can be fruitful and lead to “resolutions,” whether these resolutions are discovering the answer to an insight problem, discovering the reason something felt familiar, or discovering the word that was on the tip of our tongue. In fact, in Cleary and Schwartz (2025), we note many ways in which the subjective feeling of the TOT state drives behaviors that themselves increase the likelihood of eventually arriving at a resolution, such as generating candidate first letters of the yet-to-be-accessed target word, or simply spending more time thinking about possible words that it could be, or searching the internet for possible clues as to the word’s identity. That is, conscious experience may serve functions separate from and meta to the cognitive processes they accompany. Without getting into the philosophical debates about this topic, many have argued for a similar role of conscious experience in guiding behavior (e.g., Lau et al., 2015; Popper & Eccles, 1977).

We re-introduce the Doctrine of Concordance and why Tulving argued that it was slowing down the research program on conscious experience to urge people to consider thinking about conscious experience as an entity worth studying because as human beings we are conscious. As such, consciousness may not always be caused by the same cognitive processes that enact our behavior. By highlighting Tulving’s (1989) view that we too often assume that conscious experience always reflects the underlying cognition that drives behavior, we hope that others will take the same research path as reflected in some of the work reviewed here (Cleary et al., 2021a; Laukkonen et al., 2023; Umanath & Coane, 2020; Umanath et al., 2025; Wiley & Danek, 2024). This work has been successful precisely because the interest is in understanding the causes and consequences of conscious experience.

## Footnotes

In both psychology and philosophy, there are important differences between the use of the terms ‘conscious experience,’ ‘subjective experience,’ and ‘phenomenological experience.’ It is not the goal of this paper to explore or clarify differences in the usage of these terms. For our purposes, all three refer to the consciously-aware state of a person as compared with the non-conscious processes that we infer from behavior in experiments. Thus, we use the term conscious experience throughout, knowing that there are differences among different types of conscious experience.

“Strong in us” is a joking reference to the Star Wars movies, for those not familiar with the wordplay here.

An example of such a step-by-step problem is: Three people play a game in which one person loses and two people win each round. The one who loses must double the amount of money that each of the other two players has at that time. The three players agree to play three games. At the end of the three games, each player has lost one game and each person has $8. What was the original stake of each player? (Metcalfe & Wiebe, 1987).
